# Multimarker Approach: An Effective Tool in the Risk Stratification of Patients Admitted to the Emergency Department

**DOI:** 10.1002/jcla.70084

**Published:** 2025-08-05

**Authors:** Marilena Minieri, Carla Prezioso, Dolores Limongi, Vito N. Di Lecce, Maria Stella Lia, Alessandro Terrinoni, Alfredo Giovannelli, Gianluigi Ferrazza, Cartesio D'Agostini, Sergio Bernardini, Jacopo M. Legramante

**Affiliations:** ^1^ Department of Experimental Medicine University of Rome Tor Vergata Rome Italy; ^2^ Unit of Laboratory Medicine University Hospital Tor Vergata Rome Italy; ^3^ Laboratory of Microbiology, IRCCS San Raffaele Roma Rome Italy; ^4^ Department for the Promotion of Human Sciences and Quality of Life San Raffaele University Rome Italy; ^5^ Department of Emergency University Hospital Tor Vergata Rome Italy; ^6^ Defence Institute for Biomedical Sciences Rome Italy; ^7^ Department of Systems Medicine University of Rome Tor Vergata Rome Italy

**Keywords:** clinical decision‐making, mid‐regional‐proAdrenomedullin, mortality prediction, multimarker approach, SARS‐CoV‐2

## Abstract

**Background:**

The use of biomarkers in emergency room decision‐making has significantly increased, particularly during the COVID‐19 pandemic, due to urgent clinical needs. SARS‐CoV‐2 infection presents a spectrum of symptoms, from asymptomatic cases to severe pneumonia with respiratory failure. During the pandemic, various prognostic tools and biomarkers have been used to quickly guide patients to appropriate care upon admission. This study evaluated the effectiveness of a multimarker approach for early risk stratification of patients with confirmed SARS‐CoV‐2 infection in the Emergency Department. It aimed to determine if a combined biomarker panel could better predict COVID‐19 severity than single biomarkers, aiding in clinical decision‐making and resource management.

**Methods:**

This retrospective observational study analyzed data from 265 patients with suspected COVID‐19 admitted to the Emergency Department at the University Hospital Tor Vergata in Rome from April to December 2020. SARS‐CoV‐2 infection was confirmed by RT‐PCR swabs. Clinical features and biomarker levels were analyzed, and mortality prediction was assessed using ROC curve analysis to determine the AUC.

**Results:**

Results demonstrated that the predictive power for mortality increased when multiple biomarkers were considered together. The most comprehensive panel, combining MR‐proADM, CRP, D‐dimer, LDH, and CT score, achieved the highest accuracy (AUC: 0.866), outperforming any individual marker.

**Conclusion:**

Combining multiple biomarkers improved the prediction of disease severity over individual biomarkers. These findings suggest that using a comprehensive biomarker panel can more accurately predict SARS‐CoV‐2 severity, supporting its potential utility for early risk stratification in various emergency settings and aiding in the efficient allocation of healthcare resources.

## Graphical abstract

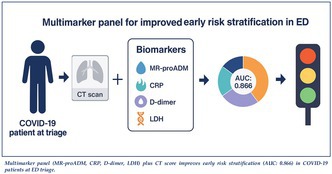

## 1 Introduction

In recent years, the use of biomarkers within the Emergency Department (ED) has increased significantly, with the attempt to help physicians in early risk stratification of critically ill patients. Biomarkers have been especially useful during the COVID‐19 pandemic to predict disease severity and support clinical decision‐making. This ability to foresee the disease progression is crucial for optimizing medical resources and determining the most appropriate clinical setting, both for excluding and including patients who need hospitalization [1, 2, 3, 4].

The SARS‐CoV‐2 infection has been a significant example of a disease with a wide clinical spectrum, ranging from asymptomatic infection to mild upper respiratory tract illness, and up to severe interstitial pneumonia with respiratory failure. The clinical course of this disease has often been unpredictable, with rapid and sudden deteriorations in patients' conditions. Consequently, various prognostic tools and biochemical markers were tested during the pandemic to direct patients toward the most appropriate clinical pathway upon admission to the emergency department.

Several biomarkers have long been used in managing other severe diseases to monitor disease progression and predict clinical outcomes. For example, D‐dimer is widely used in the diagnosis and management of venous thromboembolism and pulmonary embolism, indicating a procoagulant state in the patient [5]. C‐reactive protein (CRP) is commonly used as an inflammatory marker to detect systemic infections and acute inflammatory states, such as sepsis and chronic inflammatory diseases [6]. Lactate dehydrogenase (LDH) is an indicator of cellular damage and is used to assess severity in conditions such as acute heart failure, hematological diseases, and oncological pathologies [7, 8].

During the COVID‐19 pandemic, these same biomarkers showed significant value in predicting the severity and short‐term mortality in patients with SARS‐CoV‐2 infection. For instance, D‐dimer demonstrated significant predictive value regarding hospital mortality, indicating an intrinsic procoagulant state due to the infection. Similarly, CRP as an inflammatory marker and LDH as an indicator of cellular damage were associated with COVID‐19 severity, confirming the importance of these biomarkers in this new disease as well [9, 10].

Computed tomography (CT) also played a key role in managing COVID‐19 patients due to its high sensitivity. Recent studies have shown that CT results can be useful in the clinical management of patients with negative swab tests, but high clinical suspicion based on abnormal laboratory tests [11], as well as in the early risk stratification of patients in the emergency department [12, 13].

However, in previous studies, biomarkers have often been tested individually. Conversely, some reports have suggested that combining different biomarkers in complex panels could enhance their diagnostic and predictive value [14]. Our group demonstrated that a biomarker panel (MAPE score) based on a complete blood count has a predictive role in diagnosing SARS‐CoV‐2, thereby avoiding the swab test in patients with a low MAPE score [15].

Therefore, the objective of this study was to test whether the combined consideration of multiple biomarkers in a complex panel, specifically tested on patients with SARS‐CoV‐2, could early predict disease severity at triage in the emergency department. This approach could help optimize hospital resources and support emergency physicians in the decision‐making process regarding patient exclusion and/or inclusion, as well as in the appropriate hospital setting. Moreover, such biomarker panels could also be useful in other urgent and severe pathological conditions, overall improving the management of critical patients and enhancing the effectiveness of decision‐making processes in emergency settings.

## 2 Methods

### 2.1 Study Design

In order to test whether the combined consideration of multiple biomarkers in a complex panel could early predict disease severity at triage in the emergency department, an observational, retrospective, single‐center study was designed to analyze data from 265 consecutive patients with suspected COVID‐19 infection admitted to the Emergency Department of our University Hospital. All patients included in the study were hospitalized between April and December 2020, prior to the availability of COVID‐19 vaccines in Italy. Therefore, none of the participants had received any form of COVID‐19 vaccination at the time of SARS‐CoV‐2 infection.

The diagnosis of COVID‐19 was confirmed by a positive real‐time reverse transcription polymerase chain reaction (RT‐PCR) test from nasopharyngeal swabs and through radiological imaging, in accordance with WHO interim guidelines.

Patients aged ≥ 18 years with a positive swab test were enrolled. Epidemiological, demographic, and clinical data were extracted from electronic clinical reports (Table 1). However, due to the emergency context in which patients were admitted, lifestyle‐related variables, such as smoking status, alcohol use, or other substance consumption, were not consistently recorded and therefore were not systematically collected for this study.

**TABLE 1:** Demographic and clinical parameters.

	Overall	Survivors	Non‐survivors	*p*	NO IMV	IMV	*p*
	*N* = 265	*N* = 184	*N* = 81		*N* = 191	*N* = 74	
Age							
Years, mean (±SD)	64.0 (14.4)	60.8 (14.4)	71.7 (11.1)	< 0.001	62.0 (15.5)	68.0 (9.8)	< 0.001
Sex							
Male, *N* (%)	180 (67.9)	121 (67.2)	59 (32.8)	0.255	121 (67.2)	59 (32.8)	0.01
Female, *N* (%)	85 (32.1)	63 (74.1)	22 (25.9)		70 (82.4)	15 (17.6)	
Comorbidities							
Hypertension, *N* (%)	116 (43.8)	65 (56.0)	51 (44.0)	< 0.001	74 (63.7)	42 (36.2)	0.008
Diabetes, *N* (%)	37 (14.0)	16 (43.2)	21 (56.8)	< 0.001	18 (48.6)	19 (51.4)	0.001
Respiratory disease, *N* (%)	23 (8.7)	12 (52.2)	11 (47.8)	0.060	13 (56.6)	10 (43.4)	0.08
Malignancy, *N* (%)	13 (4.9)	7 (53.8)	6 (46.2)	0.211	6 (46.2)	7 (53.8)	0.033
Cardiovascular disease, *N* (%)	45 (17.0)	22 (48.9)	23 (51.1)	0.001	30 (66.7)	15 (33.3)	0.375
Renal disease, *N* (%)	40 (15.1)	13 (32.5)	27 (67.5)	< 0.001	15 (37.5)	25 (62.5)	0.001
Obesity, *N* (%)	12 (4.5)	7 (58.3)	5 (41.7)	0.393	6 (50.0)	6 (50.0)	0.081

Abbreviation: IMV, invasive mechanical ventilation.

CT scans were performed and then reviewed by an Emergency Department radiologist. Analyses of blood culture, sputum, urine, bronchial aspirate, and/or bronchoalveolar samples were also assessed if necessary to refine SARS‐CoV‐2 diagnosis.

The final diagnosis was provided by the emergency physician, and patient follow‐up was performed up to 45 days. This study was approved by the Local Ethics Committee of the Fondazione PTV Policlinico Tor Vergata (approval number 87/20) and was conducted in accordance with the Declaration of Helsinki. Patient informed consent was waived due to the emergency nature of dealing with this new disease.

### 2.2 Analysis of CT Images

Two experienced radiologists specializing in chest imaging independently reviewed the CT scans without access to clinical or laboratory data. Discrepancies were resolved through discussion and consensus. The images were evaluated using lung settings (width: 1500 Hounsfield Units [HU], level: −700 HU) and mediastinal settings (width: 350 HU, level: 40 HU). Recorded chest CT findings included ground‐glass opacity (GGO), crazy‐paving (CP) pattern, consolidation (CO), bronchial wall thickening, traction bronchiectasis, subpleural bands, and lesion distribution.

The number of affected lobes was noted, with predominance classified as upper (above the tracheal bifurcation), middle (between the tracheal bifurcation and the intrapulmonary vein), or lower (below the level of the intrapulmonary vein). Axial distribution was categorized as peripheral (outer third of the lung) or central (inner two‐thirds), and the pattern was considered diffuse if a clear head‐to‐tail or axial distribution was present.

Each patient underwent semi‐automated image processing to evaluate well‐ventilated lung volume, ground‐glass volume, and consolidation. Lung parenchyma modifications were analyzed using a dedicated software workstation, specifically the IntelliSpace Portal 7.0 extension (Philips, UK). Semi‐automated lung segmentation and parenchyma analysis were performed using CT‐chronic obstructive pulmonary disease (COPD) protocols.

Ground‐glass volume was quantified within the −700 HU to −300 HU range, while consolidated parenchymal volume was measured within the −300 HU to 40 HU range. The total altered lung volume was also calculated using specialized software. A semi‐quantitative CT severity score was assigned based on the extent of anatomical involvement: 0 for no involvement; 1 for < 5%; 2 for 5%–25%; 3 for 26%–50%; 4 for 51%–75%; and 5 for > 75% involvement, following the classification by Pan et al. [12].

### 2.3 Blood Sample Collection and Biomarkers Analyses

Blood samples were collected from patients at triage upon admission to the Emergency Department. For serum and plasma specimens, samples were promptly centrifuged at 4500 *g* for 5 min upon arrival at the laboratory.

Blood analyses included measurements of mid‐regional proadrenomedullin (MR‐proADM), C‐reactive protein (CRP), D‐dimer, and lactate dehydrogenase (LDH). CRP levels (normal cut‐off < 5 mg/L) and LDH levels (< 220 IU/L) were measured in serum samples using an Abbott ARCHITECT c16000 clinical chemistry analyzer (Abbott, North Chicago, USA). MR‐proADM levels (normal cut‐off < 0.55 nmol/L) were assessed with a time‐resolved amplified cryptate emission assay in EDTA plasma samples (TRACE BRAHMS MR‐proADM Kryptor, BRAHMS AG, Hennigsdorf, Germany). D‐dimer values were determined using an ACL TOP 700 Instrument (Instrumentation Laboratory Company, Werfen, Bedford, MA, USA).

### 2.4 Statistical Analysis

The primary endpoint of this study was overall in‐hospital mortality, while the secondary endpoint was the requirement for invasive mechanical ventilation (IMV). Continuous variables were expressed as mean (standard deviation) or median (interquartile range), based on data distribution, and were compared using either the Student's *t*‐test or the Mann–Whitney *U* test, as appropriate.

Categorical variables were expressed as counts and percentages and compared using the Chi‐square test or Fisher's exact test, where suitable. Associations between candidate variables and endpoints were assessed using both univariate and multivariate Cox regression analyses, with hazard ratios calculated. Comparisons between survivors and non‐survivors, as well as between patients who required ventilation and those who did not, were analyzed.

The discriminatory power of the analyzed variables to predict mortality was evaluated using receiver operating characteristic (ROC) curve analysis, with the area under the ROC curve (AUC) determined.

For the regression analysis, variables were dichotomized based on cut‐off values derived during the study's data analysis, using the Youden index from the ROC curve analysis. For each biomarker, sensitivity, specificity, negative and positive predictive values (NPV, PPV), negative and positive likelihood ratios (LR−, LR+), and odds ratios with 95% confidence intervals (CI) were reported for both mortality and IMV outcomes.

All statistical analyses were performed with SPSS software ver. 22. For the multivariate analysis, we used variables resulting statistically significant in the univariate analysis with a *p* < 0.01. Tests were considered significant if they yielded two‐tailed *p* < 0.05.

## 3 Results

### 3.1 Mortality and Mechanical Ventilation

During the study period, 81 out of 265 patients (30.6%) died, and 74 (27.9%) required invasive mechanical ventilation (IMV). Patients with a fatal outcome or requiring IMV were significantly older than survivors and non‐IMV patients, respectively (*p* < 0.001), as shown in Table 1.

Among comorbidities, hypertension was the most prevalent (43.8%) and showed a significant association with both mortality and mechanical ventilation (*p* < 0.001 and *p* = 0.008). Diabetes and chronic kidney disease were also more common among non‐survivors and IMV patients (*p* < 0.001 and *p* = 0.001), highlighting their role as risk factors for disease progression. Cardiovascular diseases were significantly associated with mortality (*p* = 0.001), though not with mechanical ventilation.

By contrast, no statistically significant differences were observed for obesity or pre‐existing respiratory diseases. These findings are detailed in Table 1 and underscore the relevance of specific comorbidities, particularly hypertension, diabetes, and renal failure, in influencing patient outcomes during the early phase of SARS‐CoV‐2 infection.

### 3.2 Biomarkers and Prognostic Accuracy

All biomarkers assessed at ED admission were significantly elevated in non‐survivors compared to survivors, and in patients requiring mechanical ventilation compared to those who did not (all *p* < 0.001). These findings are presented in Table 2. MR‐proADM exhibited the strongest association with clinical severity, with median values almost twice as high in non‐survivors and IMV patients. CRP and D‐dimer followed a similar pattern, showing markedly higher values in patients with poor outcomes. PCT and LDH also differed significantly between groups, confirming their value as indicators of systemic inflammation and tissue injury. Furthermore, CT severity scores were significantly higher in both non‐survivors and IMV patients (median 3 and 4, respectively; *p* < 0.001), reinforcing the prognostic relevance of radiological findings alongside laboratory markers. These results, summarized in Table 2, suggest that early abnormalities in inflammatory, endothelial, and coagulation biomarkers, along with CT score, can effectively stratify patients by risk at the time of ED presentation. This supports the rationale for integrating these variables into a combined panel to improve early decision‐making.

**TABLE 2:** Biomarker values at triage admission.

	Overall	Survivors	Non‐survivors	*p*	NO IMV	IMV	*p*
	*N* = 265	*N* = 184	*N* = 81		*N* = 191	*N* = 74	
MR‐proADM nmol/L, median (Q1–Q3)	0.92 (0.68–1.33)	0.80 (0.60–1.01)	1.38 (1.09–2.03)	< 0.001	0.81 (0.61–1.06)	1.35 (0.99–1.95)	< 0.001
CRP mg/L, median (Q1–Q3)	65.9 (28.50–130.0)	51.7 (18.0–92.8)	131 (75.8–204.6)	< 0.001	53 (21.0–98.0)	132 (71.0–212.0)	< 0.001
PCT ng/mL, median (Q1–Q3)	0.08 (0.04–0.20)	0.06 (0.03–0.13)	0.18 (0.095–0.40)	< 0.001	0.06 (0.03–0.13)	0.19 (0.10–0.55)	< 0.001
D‐dimer ng/mL, median (Q1–Q3)	741 (438–1446)	643 (408–1064)	1283 (687–2149)	< 0.001	666 (413–1192)	1179 (646–2047)	< 0.001
LDH U/L, median (Q1–Q3)	358 (273–489)	336 (265–432)	456 (306–589)	< 0.001	334 (257–432)	486 (331–593)	< 0.001
CT score	3 (2–4)	2 (2–4)	3 (2–5)	< 0.001	2 (2–3)	4 (2.75–5.0)	< 0.001

Abbreviations: CRP, C‐reactive protein; CT score, computed tomography score; IMV, invasive mechanical ventilation; LDH, lactate dehydrogenase; MR‐proADM, mid‐regional proadrenomedullin; PCT, procalcitonin.

### 3.3 Biomarker Panels and Predictive Power

Combining various biomarkers into panels gradually increased the prognostic accuracy for predicting both mortality and the need for mechanical ventilation, as shown in Table 3.

**TABLE 3:** Prognostic accuracy of biomarkers for different outcomes.

	Outcomes	AUC (95% CI)	Cutoff	Sensitivity (95% CI)	Specificity (95% CI)	PPV (95% CI)	NPV (95% CI)	LR+ (95% CI)	LR− (95% CI)	OR (95% CI)
MRproADM	Mortality	0.839 (0.79–0.89)	1.105	0.75 (0.64–0.84)	0.79 (0.72–0.85)	0.61 (0.54–0.68)	0.88 (0.83–0.92)	3.55 (2.62–4.82)	0.31 (0.21–0.46)	11.34 (6.12–21.01)
	IMV	0.803 (0.74–0.86)	1.105	0.74 (0.63–0.84)	0.76 (0.70–0.82)	0.55 (0.48–0.62)	0.89 (0.84–0.92)	3.15 (2.36–4.21)	0.34 (0.23–0.50)	9.39 (5.05–17.45)
CRP	Mortality	0.785 (0.73–0.84)	95.5	0.72 (0.61–0.81)	0.77 (0.70–0.83)	0.58 (0.51–0.65)	0.86 (0.81–0.90)	3.14 (2.33–4.23)	0.37 (0.26–0.52)	8.53 (4.71–15.43)
	IMV	0.775 (0.71–0.84)	63	0.84 (0.73–0.91)	0.60 (0.52–0.67)	0.45 (0.40–0.50)	0.91 (0.85–0.94)	2.08 (1.70–2.54)	0.27 (0.16–0.46)	7.65 (3.87–15.14)
D‐dimer	Mortality	0.690 (0.62–0.76)	985.5	0.65 (0.54–0.76)	0.73 (0.66–0.80)	0.52 (0.45–0.59)	0.83 (0.78–0.87)	2.46 (1.84–3.28)	0.47 (0.34–0.64)	5.26 (2.97–9.15)
	IMV	0.650 (0.58–0.72)	981.5	0.64 (0.52–0.74)	0.71 (0.64–0.77)	0.46 (0.39–0.53)	0.83 (0.79–0.87)	2.17 (1.64–2.87)	0.52 (0.38–0.71)	4.2 (2.38–7.40)
LDH	Mortality	0.675 (0.60–0.75)	439.5	0.54 (0.43–0.65)	0.79 (0.73–0.85)	0.54 (0.45–0.62)	0.80 (0.75–0.84)	2.63 (1.86–3.72)	0.58 (0.45–0.74)	4.57 (2.6–8.03)
	IMV	0.721 (0.65–0.79)	437.5	0.60 (0.47–0.71)	0.79 (0.73–0.85)	0.52 (0.44–0.61)	0.83 (0.79–0.87)	2.84 (2.03–3.96)	0.51 (0.39–0.68)	5.54 (3.10–9.89)
CT Score	Mortality	0.646 (0.57–0.72)	> 3	0.49 (0.38–0.61)	0.74 (0.67–0.80)	0.46 (0.38–0.54)	0.77 (0.73–0.81)	1.89 (1.36–2.63)	0.68 (0.54–0.86)	2.76 (1.60–4.77)
	IMV	0.719 (0.65–0.79)	> 3	0.60 (0.47–0.71)	0.77 (0.70–0.83)	0.50 (0.42–0.58)	0.83 (0.79–0.87)	2.58 (1.87–3.56)	0.53 (0.40–0.70)	4.9 (2.76–8.69)

*Note:* AUC analysis for 45‐day mortality prediction and for 28‐day IMV prediction of study population; cut‐off derived from ROC (receiver operating characteristic) using the Youden index.

Abbreviations: CRP, C‐reactive protein; CT score, computed tomography score; IMV, invasive mechanical ventilation; LDH, lactate dehydrogenase; MR‐proADM, mid‐regional‐proadrenomedullin; PCT, procalcitonin.

When MR‐proADM was combined with CRP, the area under the curve (AUC) for mortality prediction was 0.864, with a sensitivity of 75% and a specificity of 79%. The positive predictive value (PPV) was 61%, and the negative predictive value (NPV) was 88%. Similarly, the combination of MR‐proADM and D‐dimer yielded an AUC of 0.842, with a sensitivity of 79% and a specificity of 77%, resulting in a PPV of 63% and an NPV of 90%. When MR‐proADM was combined with LDH, the AUC for mortality was 0.842, with a sensitivity of 79% and a specificity of 77%. This combination provided a PPV of 60% and an NPV of 89%. The pairing of MR‐proADM with CT resulted in an AUC of 0.847, with a sensitivity of 73% and a specificity of 81%, leading to a PPV of 63% and an NPV of 87% (Table 3).

**FIGURE 1:**
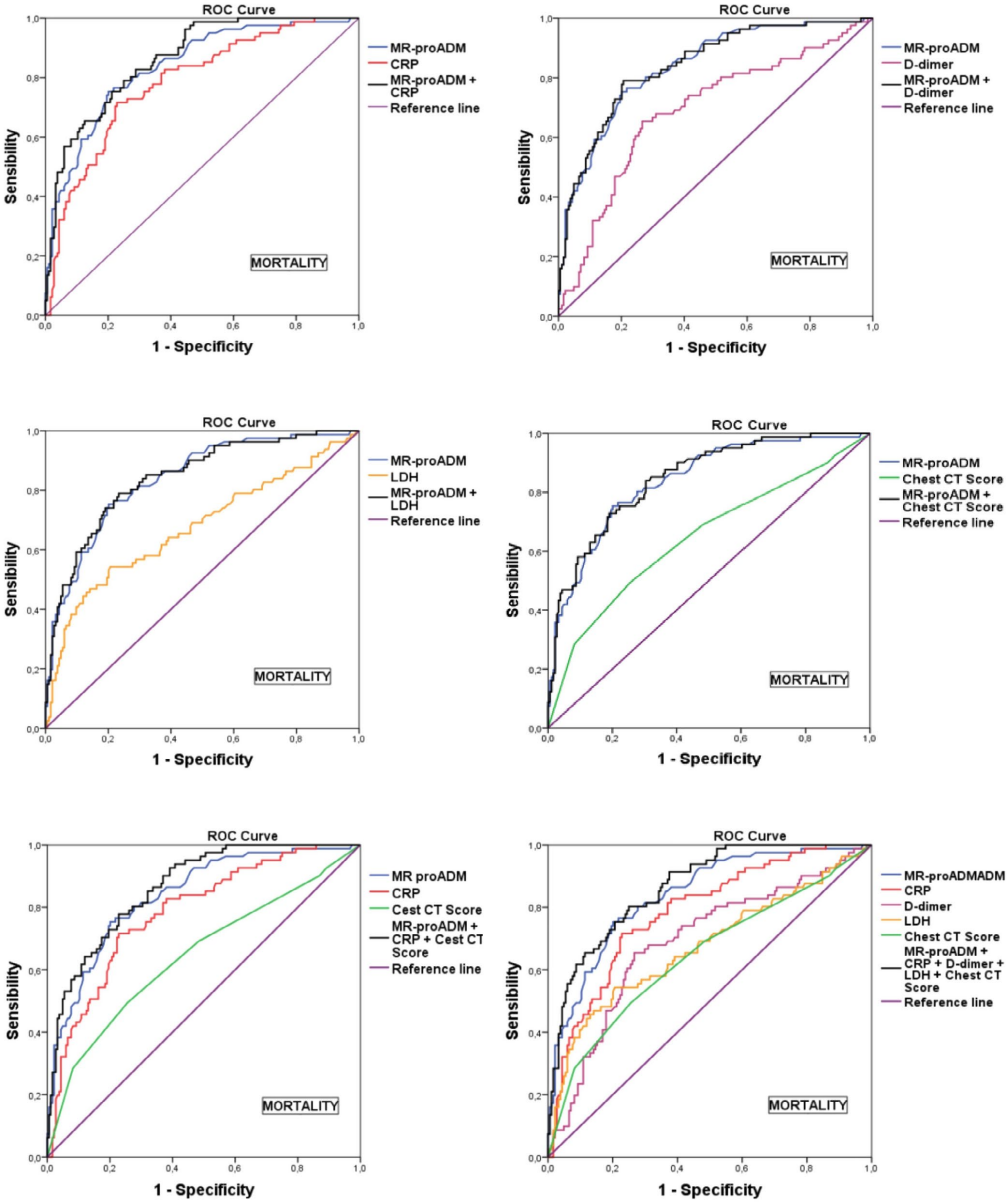
ROC curves of individual and combined biomarkers for mortality prediction. The ROC curves for various biomarkers and their combinations in predicting mortality. MR‐proADM alone and in combination with CRP, D‐dimer, LDH, and CT score were evaluated. The results demonstrate that the predictive accuracy increases with the number of biomarkers combined. Notably, the most comprehensive panel—including MR‐proADM, CRP, D‐dimer, LDH, and CT score—achieved the highest predictive power (AUC: 0.866), outperforming any individual biomarker. AUC, area under the curve; CRP, C‐reactive protein; CT, computed tomography; LDH, lactate dehydrogenase; MR‐proADM, mid‐regional proadrenomedullin.

**FIGURE 2:**
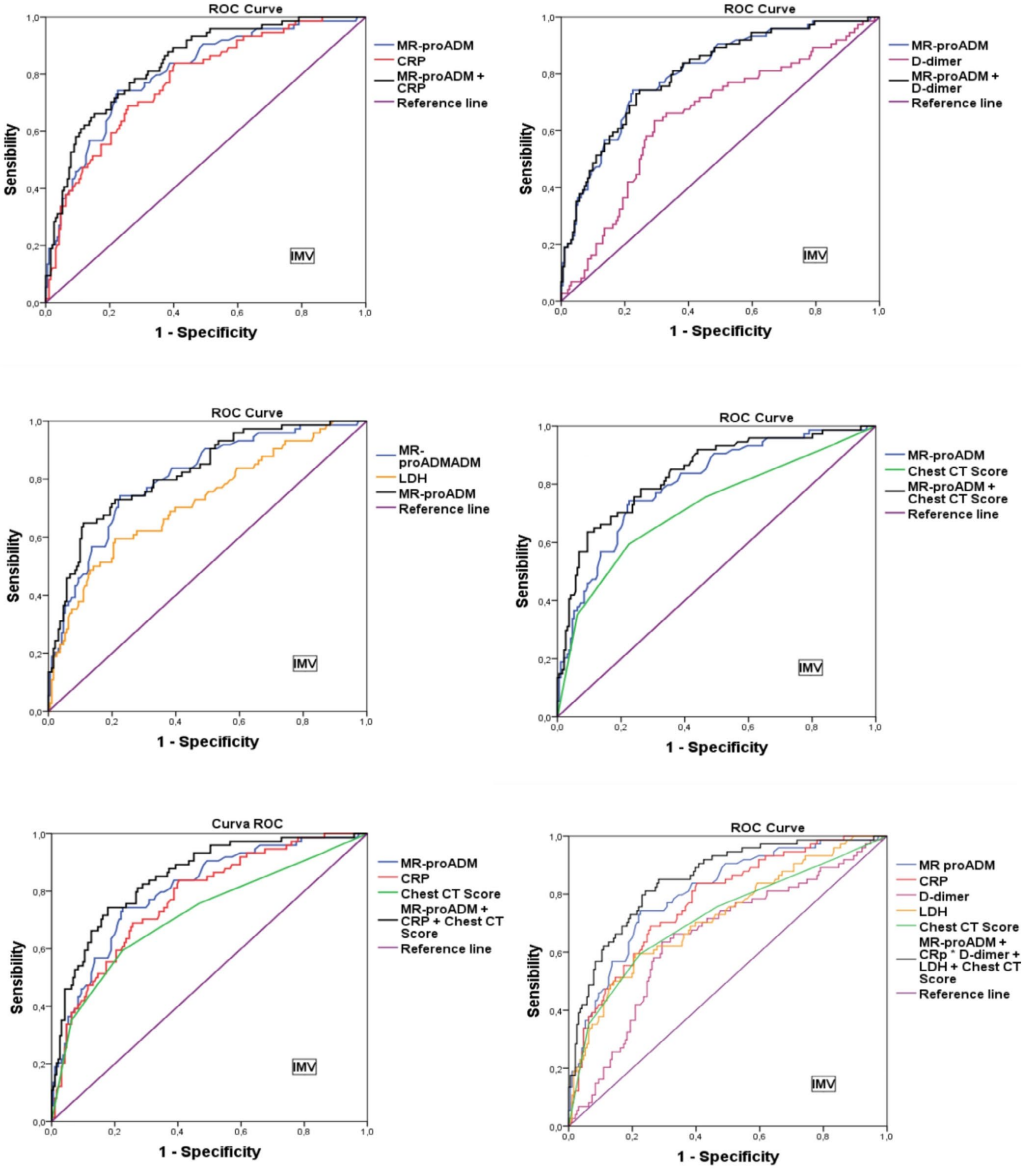
ROC curves for predicting the need for mechanical ventilation (MV) using individual and combined biomarkers. The ROC curves assessing the predictive performance of various biomarkers and their combinations for the need for mechanical ventilation. MR‐proADM was analyzed alone and in combination with CRP, D‐dimer, LDH, and CT score. As seen with mortality prediction, the accuracy of prediction improved as more biomarkers were included. The most comprehensive panel—including MR‐proADM, CRP, D‐dimer, LDH, and CT score—achieved the highest overall performance (AUC: 0.866), providing the best sensitivity and negative predictive value. AUC, area under the curve; CRP, C‐reactive protein; CT, computed tomography; LDH, lactate dehydrogenase; MR‐proADM, mid‐regional pro‐adrenomedullin; MV, mechanical ventilation.

Including additional biomarkers further improved predictive accuracy. The combination of MR‐proADM, CRP, and CT produced an AUC of 0.866, with a sensitivity of 78% and a specificity of 77%. The PPV for this panel was 60%, and the NPV was 89%. The most comprehensive panel, which included MR‐proADM, CRP, LDH, D‐dimer, and CT, also showed an AUC of 0.866, but with a slightly higher sensitivity of 80% and a specificity of 75%. This panel provided a PPV of 59% and an NPV of 90% (Table 4, Figures 1 and 2).

**TABLE 4:** Prognostic accuracy of different combinations of biomarkers for different outcomes.

	Outcomes	AUC (95% CI)	Sensitivity (95% CI)	Specificity (95% CI)	PPV (95% CI)	NPV (95% CI)	LR+ (95% CI)	LR− (95% CI)	OR (95% CI)
MR‐pro ADM + CRP	Mortality	0.864 (0.82–0.91)	0.75 (0.64–0.84)	0.79 (0.72–0.84)	0.61 (0.54–0.68)	0.88 (0.83–0.91)	3.55 (2.62–4.82)	0.31 (0.21–0.46)	11.34 (6.12–21.01)
	IMV	0.837 (0.79–0.89)	0.66 (0.54–0.77)	0.85 (0.79–0.90)	0.63 (0.54–0.71)	0.87 (0.82–0.90)	4.36 (3.0–6.33)	0.40 (0.29–0.55)	10.95 (5.87–20.42)
MR‐pro ADM + D‐dimer	Mortality	0.842 (0.79–0.89)	0.79 (0.69–0.87)	0.79 (0.73–0.85)	0.63 (0.55–0.70)	0.90 (0.85–0.93)	3.83 (2.82–5.19)	0.26 (0.17–0.41)	14.46 (7.6–27.51)
	IMV	0.804 (0.75–0.86)	0.74 (0.63–0.84)	0.75 (0.69–0.81)	0.54 (0.47–0.61)	0.88 (0.84–0.92)	3.02 (2.28–4.0)	0.34 (0.23–0.51)	8.87 (4.79–16.43)
MR‐pro ADM + LDH	Mortality	0.842 (0.79–0.89)	0.79 (0.69–0.87)	0.77 (0.70–0.83)	0.60 (0.53–0.66)	0.89 (0.84–0.93)	3.38 (2.54–4.49)	0.27 (0.18–0.42)	12.35 (6.55–23.28)
	IMV	0.822 (0.77–0.88)	0.65 (0.53–0.76)	0.89 (0.84–0.93)	0.70 (0.60–0.78)	0.87 (0.83–0.90)	5.90 (3.81–9.13)	0.39 (0.29–0.54)	14.95 (7.74–28.86)
MR‐pro ADM + CT	Mortality	0.847 (0.80–0.90)	0.73 (0.62–0.82)	0.81 (0.75–0.86)	0.63 (0.55–0.70)	0.87 (0.83–0.91)	3.83 (2.76–5.31)	0.34 (0.23–0.48)	11.42 (6.19–21.07)
	IMV	0.837 (0.78–0.89)	0.64 (0.52–0.74)	0.91 (0.86–0.94)	0.72 (0.62–0.81)	0.87 (0.83–0.90)	6.74 (4.20–10.8)	0.40 (0.30–0.55)	16.73 (8.49–32.96)
MR‐pro ADM + CRP + CT score	Mortality	0.865 (0.82–0.91)	0.78 (0.67–0.86)	0.77 (0.70–0.83)	0.60 (0.53–0.67)	0.89 (0.84–0.92)	3.41 (2.55–4.55)	0.29 (0.19–0.44)	11.83 (6.32–22.15)
	IMV	0.850 (0.80–0.90)	0.74 (0.63–0.84)	0.82 (0.76–0.87)	0.62 (0.54–0.70)	0.89 (0.85–0.92)	4.18 (2.99–5.82)	0.31 (0.21–0.46)	13.37 (7.05–25.35)
MR‐pro ADM + CRP + LDH + D‐dimer + CT score	Mortality	0.866 (0.82–0.91)	0.80 (0.70–0.88)	0.75 (0.68–0.81)	0.59 (0.52–0.65)	0.90 (0.85–0.93)	3.21 (2.44–4.22)	0.26 (0.17–0.41)	12.19 (6.42–23.13)
	IMV	0.852 (0.80–0.90)	0.81 (0.70–0.89)	0.76 (0.69–0.82)	0.57 (0.50–0.63)	0.91 (0.87–0.94)	3.37 (2.56–4.43)	0.25 (0.15–0.40)	13.51 (6.92–26.39)

*Note:* AUC analysis for 45‐day mortality prediction and for 28‐day IMV prediction of study population; cut‐off derived from ROC (receiver operating characteristic) using the Youden index.

Abbreviations: CRP, C‐reactive protein; CT score, computed tomography score; IMV, invasive mechanical ventilation; LDH, lactate dehydrogenase; MR‐proADM, mid‐regional‐proadrenomedullin; PCT, procalcitonin.

The most important statistical index for emergency physicians is the negative predictive value (NPV). The biomarker panels demonstrate that considering a greater number of biomarkers increases the NPV, thereby improving the predictive accuracy for both mortality and the need for mechanical ventilation.

## 4 Discussion

The clinical utility of biomarkers in the Emergency Department (ED) has been well established, particularly in the context of acute infections such as pneumonia, where their rapid turnaround and widespread availability support timely decision making [15, 16, 17].

Building on this evidence, our previous studies have demonstrated that MR‐proADM is effective in the risk stratification of patients with community‐acquired pneumonia (CAP), helping to determine the appropriate level of care promptly [18, 19]. The COVID‐19 pandemic has further underscored the importance of optimizing emergency department resources and prioritizing hospitalization for seriously ill patients to ensure appropriate care levels.

Prior research has indicated that biomarkers, particularly MR‐proADM, can predict outcomes in hospitalized patients with COVID‐19‐related pneumonia [1, 2]. Our group has shown that MR‐proADM, combined with other biomarkers, can assist emergency physicians in predicting outcomes for COVID‐19 patients [3, 20, 21]. However, these biomarkers have typically been tested individually [14]. It has been suggested that panels of different biomarkers might offer better predictive power than single biomarkers alone [14].

Despite this, there has been a lack of data on the effectiveness of biomarker panels in predicting clinical severity for patients with SARS‐CoV‐2 infections admitted to the ED.

This study, to our knowledge, is the first to evaluate the ability of panels consisting of more than one biomarker in the early risk stratification of COVID‐19 patients in the Emergency Department. Our findings clearly show that several biomarkers considered together can predict the severity of SARS‐CoV‐2 infection better than a single biomarker at a time. Combining multiple biomarkers into a single panel significantly enhances prognostic power. The area under the curve (AUC) for mortality prediction increased progressively with the addition of each biomarker, indicating improved discriminatory power. The most comprehensive panel, which included MR‐proADM, CRP, LDH, D‐dimer, and CT, demonstrated the highest predictive accuracy with an AUC of 0.866, a sensitivity of 80%, and a specificity of 75%.

The negative predictive value (NPV) is particularly crucial for emergency physicians, as it helps in ruling out patients unlikely to require intensive interventions, thereby reducing emergency department overcrowding. Our data showed that the NPV increased with the inclusion of more biomarkers, emphasizing the reliability of these panels in ruling out severe disease in COVID‐19 patients. This is vital for efficient patient management and optimal allocation of hospital resources.

Although the data analyzed in our study were collected during the first wave of the pandemic, when the ancestral SARS‐CoV‐2 strain was predominant, we believe that our findings maintain their clinical relevance in subsequent phases. The biomarkers used in our multimarker panels (MR‐proADM, CRP, D‐dimer, and LDH) are not variant‐specific, but instead reflect host pathophysiological responses such as systemic inflammation, endothelial dysfunction, and coagulation abnormalities. These mechanisms are shared across different Variants of Concern, including Alpha, Delta, and Omicron.

Therefore, the predictive validity of our biomarker‐based approach can be considered robust and generalizable, not only across different SARS‐CoV‐2 variants but also potentially applicable to other acute viral respiratory infections that share similar clinical trajectories. This strengthens the potential utility of multimarker panels as stable and adaptable tools for emergency physicians, especially in scenarios of emerging infectious diseases where specific virological data may not be immediately available.

Age was modeled as a continuous variable to allow a more precise, data‐driven representation of its association with clinical outcomes and to avoid arbitrary thresholds that may reduce statistical power.

Notably, all the biomarkers involved in our panels are widely utilized and readily available in most hospital laboratories. Additionally, chest computed tomography (CT), which is included in our panels, has become a gold standard in diagnosing COVID‐19 in emergency departments due to its high sensitivity and diagnostic accuracy [11, 12, 13].

The widespread availability and established use of these tools make it feasible to implement these panels in routine clinical practice for the risk stratification of patients with vital infections in emergency settings.

The implications of these findings extend beyond COVID‐19, potentially impacting the management of general infectious diseases and other emergency conditions. The validated biomarker panels could play a significant role in early risk stratification and management of critically ill patients, offering a robust tool for emergency physicians. By providing a reliable method to predict disease severity, these panels can help optimize the use of medical resources, improve patient outcomes, and support more effective decision‐making in emergency care.

Interestingly, our panel included a score accounting for chest tomography, which represents a gold standard in the diagnostic procedure of COVID‐19 patients. This study extends previous results, supporting emergency physicians in deciding the adequate clinical setting according to the possible need for ventilation, thus contributing to optimizing hospital resources.

However, it is essential to recognize that biomarkers might oversimplify the interpretation of important variables. Consequently, they should be considered a valid aid, although not a replacement for clinical judgment and the right consideration of validated severity scores.

Our study demonstrates the enhanced predictive power of combining multiple biomarkers into panels for early risk stratification of COVID‐19 patients in the ED. These findings support the use of biomarker panels to predict disease severity better than individual biomarkers, aiding emergency physicians in making informed decisions about patient care and resource allocation.

## 5 Conclusions

In conclusion, the experience gained during the COVID‐19 pandemic has highlighted the critical need for advanced diagnostic and prognostic tools in the Emergency Department. Our study underscores the importance of combining multiple biomarkers into comprehensive panels to enhance early risk stratification. This approach not only optimizes resource allocation and improves patient outcomes in COVID‐19 but also holds significant potential for managing a wide range of infectious diseases and emergency conditions. By incorporating these validated biomarker panels into routine practice, healthcare systems can better prepare for future challenges, ensuring timely and appropriate care for critically ill patients.

## Ethics Statement

The study was conducted in accordance with the Declaration of Helsinki and approved by the local Ethics Committee at Tor Vergata University Hospital (approval number 87/20; 26 May 2020).

## Consent

Written informed consent was waived because of the rapid spread of this infectious disease.

## Conflicts of Interest

The authors declare no conflicts of interest.

## Data Availability

The data that support the findings of this study are available on request from the corresponding author. The data are not publicly available due to privacy or ethical restrictions.
